# Roles of Toric intraocular Lens implantation on visual acuity and astigmatism in glaucomatous eyes treated with iStent and cataract surgery

**DOI:** 10.1186/s12886-022-02707-1

**Published:** 2022-12-14

**Authors:** Sho Ichioka, Akiko Ishida, Yuji Takayanagi, Kaoru Manabe, Masato Matsuo, Masaki Tanito, Masaki Tanito

**Affiliations:** Department of Ophthalmology, Shimane University Faculty of Medicine, 89-1 Enya, Izumo, 693-8501 Shimane Japan

**Keywords:** iStent trabecular micro-bypass, Surgically induced astigmatism, Vector analysis, Minimally invasive glaucoma surgery (MIGS), Visual acuity, Primary open angle glaucoma, Exfoliation glaucoma

## Abstract

**Background:**

To assess the efficacy of toric intraocular lenses (IOLs) in combined cataract and minimally invasive glaucoma surgery (MIGS), visual and refractive outcomes were compared between eyes implanted with non-toric and toric IOLs during iStent triple procedures.

**Methods:**

In this retrospective study, open angle glaucoma eyes with preexisting corneal astigmatism of -1.5 diopter (D) or more and implanted with non-toric (n = 9) or toric (n = 9) IOLs were included. The main outcome measures were the intergroup difference in the uncorrected visual acuity (UCVA) and refractive astigmatism at 3 months postoperatively.

**Results:**

Preoperatively, the logarithm of the minimum angle of resolution (logMAR) UCVAs and refractive astigmatism were equivalent between the groups. Postoperatively, the logMAR UCVA (non-toric, 0.45 ± 0.31; toric, 0.14 ± 0.15; *P* = 0.021) was significantly better and the refractive astigmatism (non-toric, -2.03 ± 0.63 D; toric, -0.67 ± 0.53 D; *P* = 0.0014) significantly less in the toric group. The toric group had postoperative improvements in the logMAR UCVA (-0.21, *P* = 0.020) and refractive astigmatism (+ 1.72 D, *P* = 0.0039). Vector analyses showed the postoperative centroid magnitude and confidence eclipses of refractive astigmatism was less in the toric group (0.47 D at 173°±0.73D) than the non-toric group (1.10 D at 2°±1.91D). Postoperatively, 78% of eyes in the toric group had 1.0 D or less refractive astigmatism compared with 11% in the non-toric group. Surgically induced astigmatism (non-toric, 0.18 D at 65°; toric, 0.29 D at 137°) did not differ between groups.

**Conclusion:**

Use of toric IOLs is a reasonable option for better visual outcomes when the combined cataract and iStent surgery is performed in glaucomatous eyes with corneal astigmatism.

## Introduction

Postoperative decrease of visual acuity due to surgically-induced astigmatism (SIA) is one of the major demerits of trabeculectomy (LEC) [1]. On the other hand, combined cataract surgery and minimally-invasive glaucoma surgery (MIGS) using iStent trabecular micro-bypass stent were reported to be refractively neutral [2, 3]. We previously reported that another MIGS procedure, Tanito microhook trabeculotomy (TMH) associated with remarkable less SIA than filtration surgeries such as LEC and ExPRESS shunt [4]. Good predictability and small amount of SIA prompted us to use toric intraocular lens (IOL) during the combined cataract extraction and ab interno MIGS procedures including TMH and iStent for correction of corneal astigmatism. Other than cataract surgery alone, toric IOLs have been used during combined cataract surgery and small-gauge vitrectomy[5, 6]; we have reported the roles of toric IOLs during TMH [7, 8], however only a recent study reported the use of toric IOLs during iStent [9]. In this study, we compared the visual acuity and refractive status between glaucomatous eyes that implanted with non-toric and toric IOLs during combined iStent. To the best of knowledge, comparison between non-toric and toric IOLs after iStent was unique in the literature.

## Subjects and methods

The study design and methods for statistical analyses were followed our previous works that tested the the roles of toric IOLs during TMH [7, 8].

## Subjects

This retrospective study included 18 eyes of 18 subjects implanted with a non-toric IOL (Vivinex iSert XY1, Hoya, Tokyo, Japan; n = 9) or a toric IOL (Vivinex Toric XY1, Hoya; n = 9) during iStent and simultaneous small-incision cataract surgery performed at Shimane University Hospital. Since we started to use the toric IOL model in July 2019, the subjects who met the inclusion criteria were selected from our departments’ glaucoma database in a chronological order of surgical date from July 2019 ascendingly for non-toric group and descendingly for toric group to include 9 eyes in each of surgical group.

The study adhered to the tenets of the Declaration of Helsinki; the institutional review board (IRB) of Shimane University Hospital reviewed and approved the research. Preoperatively, all subjects provided written informed consent for surgery; however, the IRB approval did not require that each patient provide written informed consent for publication; instead, the study protocol was posted at the study institutions to notify participants about the study. Only anonymous data were used in the statistical analyses. The inclusion criteria included the following: patients underwent the surgery performed by the same surgeons (MT or MM); the presence of preoperative corneal astigmatism measured by keratometry exceeded − 1.5 D; a target postoperative spherical refractive error of 0 D; absence of visually significant ocular diseases other than glaucoma and cataract; data included uncorrected visual acuity (UCVA), best-corrected VA (BCVA), refractive error for BCVA, spherical equivalent refractive error (SERE), IOP, number of glaucoma medications, and keratometric corneal astigmatism recorded preoperatively and 3 months (2–4 months) postoperatively; no intraoperative complications; and postoperative decimal BCVA of 0.8 or better. If both eyes of a patient were eligible, the eye that initially underwent surgery was included. The BCVA measured using a decimal VA chart was converted to the logarithm of the minimum angle of resolution (logMAR) VA. The IOP was measured by Goldmann applanation tonometry. The keratometry was recorded at the central 3-millimeter diameter by autorefract-keratometry (TonoRef III, Nidek, Gamagori, Japan).

## Surgical Procedure

The surgical procedure was performed through two corneal side ports as reported previously [10, 11]. Before iStent implantation, phacoemulsification cataract surgery was performed through a 2.2-mm-wide clear corneal incision created at the 9 to 10 o’clock position (i.e., temporal incision for the right eye and nasal incision for the left eye); a one-piece soft acrylic IOL (non-toric or toric) was inserted into the capsular bag through the same clear corneal incision. After then, under the observation using a Swan-Jacob gonioprism lens (Ocular Instruments, Bellevue, WA), the first-generation iStent device (GTS100R for right eyes and GTS100L for left eyes, Glaukos Japan, Tokyo, Japan) was implanted into Schlemm’s canal through the TM at the inferonasal quadrant. During the implantation, viscoelastic material (1% sodium hyaluronate, Provisc, Alcon Japan, Tokyo, Japan) was used to maintain the anterior chamber. In cases implanted with toric IOLs, the axes of the IOLs were aligned by referencing the positions of the episcleral/conjunctival vessels, the viscoelastic material was aspirated, and the corneal ports were closed by corneal stromal hydration. No miotics was used during the surgery; alignment of IOL axis was performed during the viscoelastic aspiration. At the end of surgery, a steroid (2 mg of betamethasone sodium phosphate, Rinderone, Shionogi Pharmaceutical) was injected subconjunctivally and 0.3% ofloxacin ointment (Tarivid, Santen Pharmaceutical) was applied. Finally, 1.5% levofloxacin (Nipro, Osaka Japan) and 0.1% betamethasone (Sanbetason, Santen Pharmaceutical) were applied topically four times daily for 3 to 4 weeks postoperatively in all cases.

## Lens Power calculation

The IOL spherical power was calculated in all patients using Barrett’s formula using the refraction and axial length measured by OA2000 (Tomey, Nagoya, Japan), and the targeted refraction was emmetropia. Preoperatively, absence of remarkable irregular astigmatism was confirmed by using a corneal topography map of OA2000. In the toric IOL group, the IOL cylindrical power and alignment axis were calculated using a web-based toric IOL calculator (HOYA toric calculator, https://www.hoyatoric.com). Based on the information provided by the manufacturer, the XY1AT3 (0 eye), XY1AT4 (3 eyes), XY1AT5 (4 eyes), XY1AT6 (1 eye), and XY1AT7 (1 eye) toric IOLs were selected for use when the preoperative corneal astigmatism levels were approximately 1.04, 1.56, 2.08, 2.60, and 3.12 D, respectively.

## Sample size calculation

The main outcome measures were intergroup (i.e., between the non-toric and toric groups) comparisons of the UCVA and residual refractive astigmatism at postoperative month 3. Because previous reports on the postoperative refraction of the toric IOL models used in our study had been unavailable in the literature, we referred to the data from another toric IOL model (i.e., AcrySof toric IOL, Alcon Inc.).[12, 13] In a previous report that compared the absolute residual refractive astigmatism after cataract surgery between eyes implanted with the AcrySof toric and non-toric IOLs in subjects with cataracts and pre-existing corneal astigmatism, the mean absolute residual refractive cylinder was 0.59 D after toric IOL implantation and 1.22 D after non-toric IOL implantation [12]. If the difference in the residual refractive cylinder between the two groups was set at 0.33 D [13] with a significance level of 0.05 and a power of 0.8, an estimated sample size of at least six eyes in each group was required to detect significant differences between the two groups. Considering the possibility of increasing the standard deviation, 9 eyes each were enrolled in each group.

## Analysis of astigmatism parameters

In addition to the arithmetic analyses of the astigmatism magnitudes, the astigmatic data were analyzed using American Society of Cataract and Refractive Surgery’s Astigmatism Double Angle Plot Tool version 1.1.0 (https://ascrs.org/tools/astigmatism-double-angle-plot-tool) based on the vector analysis algorithm [14]. Using this tool, the distributions of the preoperative and postoperative corneal astigmatism in the two IOL groups were visualized. The degrees of SIA were calculated in each group from the keratometric values obtained preoperatively and 3 months postoperatively using the SIA Calculator Version 2.1 developed by Drs. Saurabh Sawhney and Aashima Aggarwal (http://www.insighteyeclinic.in/SIA_calculator.php) [15].

## Statistical analyses

The age, preoperative and postoperative data from the IOPs, number of glaucoma medications, UCVA, BCVA, refractive spherical error, refractive astigmatism, SERE, and corneal astigmatism were compared between the two groups using the Mann-Whitney U-test. Sex and glaucoma type were compared using Fisher’s exact probability test. In each group, the changes in the IOPs, number of glaucoma medications, UCVA, BCVA, refractive spherical error, refractive astigmatism, SERE, and corneal astigmatism were calculated between preoperatively and postoperatively using Wilcoxon’s signed-rank text. *P* < 0.05 was considered significant. All statistical analyses were performed using the JMP Pro version 15.2.1 statistical software (SAS Institute, Inc., Cary, NC, USA).

## Results

Subject age, sex, glaucoma types, preoperative and postoperative IOPs, and medication scores were equivalent between the two groups (Table 1). Compared to preoperatively, the IOPs and medications were not significantly changed postoperatively in both groups (Table 1).

**Table 1:** Demographic subject data, intraocular pressures, and medication scores

	Non-Toric	Toric	*P* value
No.			
Eyes/Subjects	9/9	9/9	
Age (years)			
Mean ± SD	82.6 ± 8.2	78.3 ± 6.4	0.20†
95% CI	76.3, 88.8	73.4, 83.3	
Sex, no. (%)			
Male	6 (67)	5 (56)	1.0‡
Female	3 (33)	4 (44)	
Glaucoma type, no. (%)			
POAG	7 (78)	9 (100)	0.47‡
EXG	2 (22)	0 (0)	
Preoperative IOP (mmHg)			
Mean ± SD	14.8 ± 4.0	15.0 ± 3.7	0.72†
95% CI	11.7, 17.8	12.1, 17.9	
Postoperative IOP (mmHg)			
Mean ± SD	12.1 ± 3.7	13.6 ± 1.7	0.27†
95% CI	9.2, 15.0	12.3, 14.8	
Changes in IOP (mmHg)			
Mean ± SE	-2.7 ± 1.8	-1.4 ± 1.0	
95% CI	-6.8, 1.4	-3.8, 0.9	
*P* values$	0.19	0.19	
Preoperative medications			
Mean ± SD	2.3 ± 1.9	2.2 ± 1.2	0.74†
95% CI	0.8–3.8	1.3–3.1	
Postoperative medications			
Mean ± SD	1.6 ± 0.9	1.2 ± 1.1	0.52†
95% CI	0.3–2.2	0.4–2.1	
Changes in medications			
Mean ± SE	-0.8 ± 0.5	-1.0 ± 0.5	
95% CI	-1.9, 0.4	-2.2–0.2	
*P* values$	0.25	0.13	

†P values calculated by Mann-Whitney U test between non-toric and toric groups. ‡P values calculated by Fisher’s exact probability test between non-toric and toric groups. $P values calculated by Wilcoxon’s signed-rank test between pre- and post-operative values in each non-toric or toric group. The postoperative values are collected 3 months postoperatively. SD, standard deviation; 95% CI, 95% confidence interval; POAG, primary open-angle glaucoma; EXG, exfoliation glaucoma; IOP, intraocular pressure; SE, standard error.

Preoperatively, the UCVA, BCVA, refractive spherical error, refractive astigmatism, and SERE were equivalent between the groups (Table 2). Postoperatively, the UCVAs were significantly better in the toric group (0.45 logMAR) than the non-toric group (0.14 logMAR, *P* = 0.021), while the BCVAs were equivalent between the groups (Table 2). Postoperatively, the refractive astigmatism values were significantly smaller in the toric group (-0.67 D) than the non-toric group (-2.03 D, *P* = 0.0014), this accompanied significantly smaller refractive spherical error values in the toric group (+ 0.19 D) than the non-toric group (+ 1.03 D, p = 0.036), while the SEREs were equivalent between the groups (Table 2). The BCVAs improved in both groups, while postoperative improvements in the UCVA (-0.21 logMAR, *P* = 0.020) and refractive astigmatism values (+ 1.72 D, *P* = 0.0039) occurred only in the toric group (Table 2).

**Table 2:** Visual acuities and refractive errors

	Non-Toric	Toric	*P* value†
Preoperative UCVA (logMAR)			
Mean ± SD	0.43 ± 0.24	0.34 ± 0.11	0.44
95% CI	0.25, 0.61	0.26, 0.43	
Postoperative UCVA (logMAR)			
Mean ± SD	0.45 ± 0.31	0.14 ± 0.15	0.021
95% CI	0.21, 0.69	0.02, 0.25	
Change in UCVA (logMAR)			
Mean ± SE	0.02 ± 0.06	-0.21 ± 0.06	
95% CI	-0.12, 0.17	-0.33, -0.08	
*P* values$	1.00	0.020	
Preoperative BCVA (logMAR)			
Mean ± SD	0.20 ± 0.13	0.19 ± 0.15	1.00
95% CI	0.10, 0.30	0.08, 0.31	
Postoperative BCVA (logMAR)			
Mean ± SD	0.04 ± 0.06	0.02 ± 0.06	0.35
95% CI	0.00, 0.09	-0.02, 0.06	
Change in BCVA (LogMAR)			
Mean ± SE	-0.16 ± 0.04	-0.17 ± 0.05	
95% CI	-0.24, -0.08	-0.28, -0.07	
*P* values$	0.0078	0.016	
Preoperative refractive spherical error (D)			
Mean ± SD	1.11 ± 0.91	1.33 ± 1.85	0.72
95% CI	0.41, 1.81	-0.09, 2.75	
Postoperative refractive spherical error (D)			
Mean ± SD	1.03 ± 0.75	0.19 ± 0.56	0.036
95% CI	0.45, 1.61	-0.23, 0.62	
Change in refractive spherical error (D)			
Mean ± SE	-0.08 ± 0.35	-1.14 ± 0.62	
95% CI	-0.90, 0.73	-2.57, 0.30	
P values$	0.77	0.16	
Preoperative refractive astigmatism (D)			
Mean ± SD	-2.25 ± 0.68	-2.39 ± 0.50	0.71
95% CI	-1.72, -2.78	-2.00, -2.77	
Postoperative refractive astigmatism (D)			
Mean ± SD	-2.03 ± 0.63	-0.67 ± 0.53	0.0014
95% CI	-2.51, -1.54	-1.07, -0.26	
Change in refractive astigmatism (D)			
Mean ± SE	0.22 ± 0.32	+ 1.72 ± 0.25	
95% CI	-0.52, 0.97	1.14, 2.30	
*P* values$	0.45	0.0039	
Preoperative SERE (D)			
Mean ± SD	0.01 ± 0.59	-0.14 ± 0.41	0.40
95% CI	-0.44, 0.47	-0.45, 0.17	
Postoperative SERE (D)			
Mean ± SD	-0.01 ± 0.87	0.14 ± 1.69	0.69
95% CI	-0.68, 0.66	-1.16, 1.43	
Change in SERE (D)			
Mean ± SE	0.03 ± 0.31	-0.28 ± 0.59	
95% CI	-0.68, 0.73	-1.63, 1.08	
*P* values$	0.81	0.61	

†P values calculated by Mann-Whitney U test between non-toric and toric groups. $P values calculated by Wilcoxon’s signed-rank test between pre- and post-operative values in each non-toric or toric group. The postoperative values are collected 3 months postoperatively.

UCVA, uncorrected visual acuity; logMAR, logarithm of the minimum angle of resolution; SD, standard deviation; 95% CI, 95% confidence interval; SE, standard error; BCVA, best-corrected visual acuity; D, diopter; SERE, spherical equivalent refractive error.

**Fig. 1:**
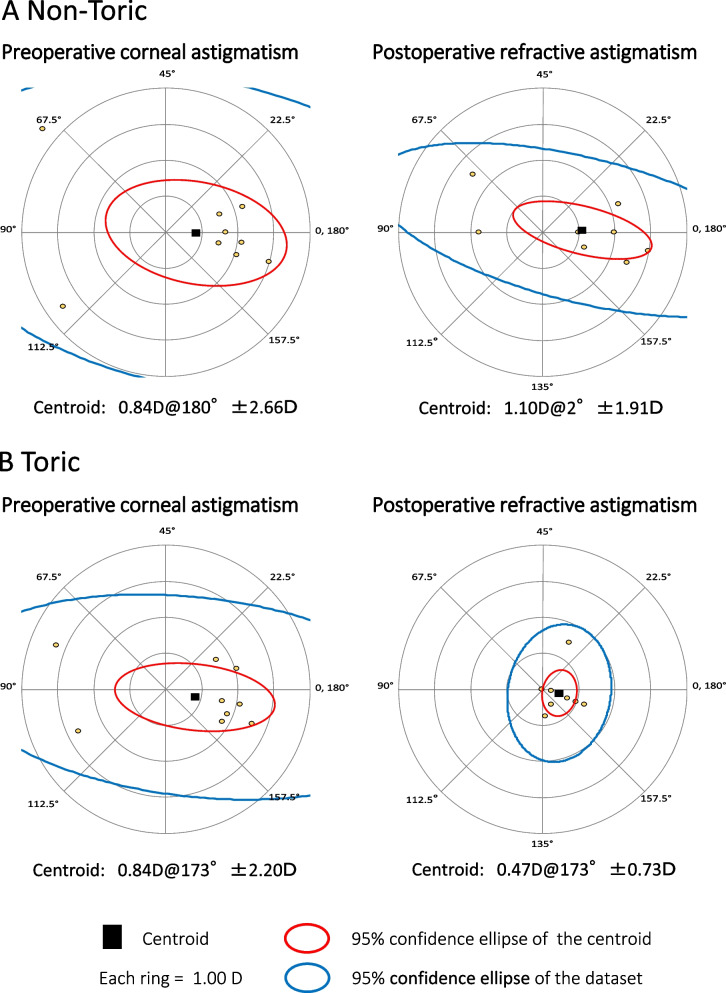
Double-angle plots for preoperative corneal and postoperative refractive astigmatism values in the non-toric (A) and toric (B) groups. *D* indicates diopters

**Fig. 2:**
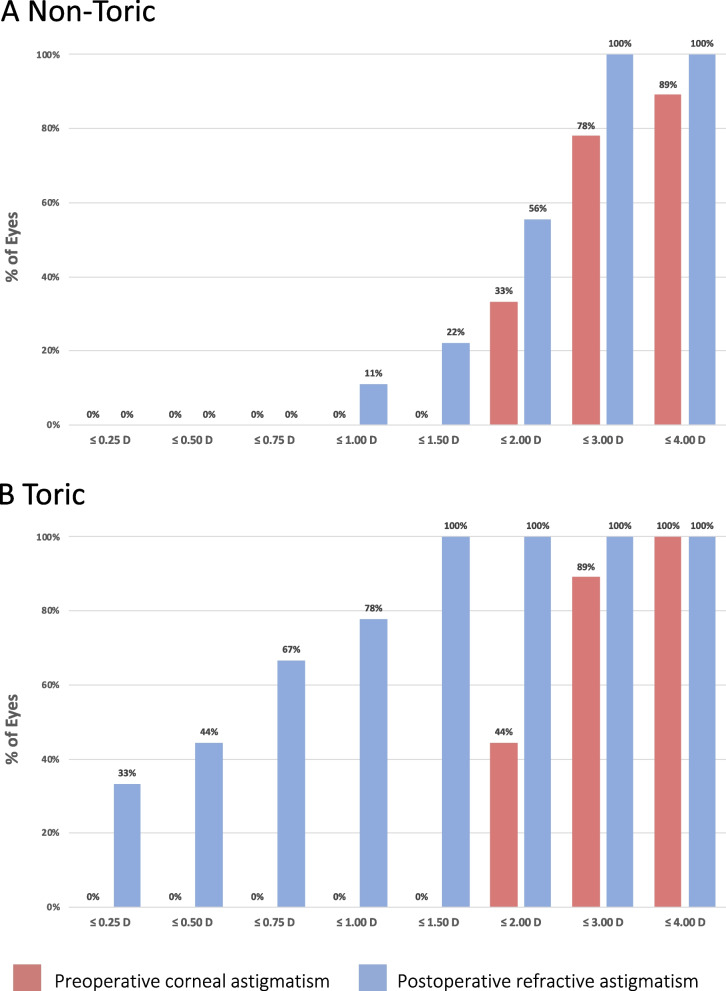
Cumulative histogram of preoperative corneal and postoperative refractive astigmatism values in the non-toric (A) and toric (B) groups. *D* indicates diopters

The preoperative and postoperative corneal astigmatism levels were equivalent between the groups; the arithmetic mean of the corneal astigmatism magnitude was unchanged postoperatively in both groups (Table 3). Vector analyses showed that the SIAs in both groups (non-toric, 0.18 D at 65°; toric, 0.29 D at 137°) did not differ markedly (Table 3). Double-angle plots for the preoperative corneal astigmatism and postoperative refractive astigmatism in both groups are shown in Fig. 1. By the vector analysis, the preoperative centroid magnitudes in the non-toric (0.84 D at 180°±2.66D) (Fig. 1 A) and toric (0.84 D at 173°±2.20D) (Fig. 1B) groups were equivalent. Postoperatively, the centroid magnitude and confidence eclipses in the toric group (0.47 D at 173°±0.73D) (Fig. 1B) were obviously smaller than in the non-toric group (1.10 D at 2°±1.91D) (Fig. 1 A). Postoperatively, 78% of eyes in the toric group had refractive astigmatism of 1.0 D or less (Fig. 2B), while 11% of eyes in the non-toric group had refractive astigmatism of 1.0D or less (Fig. 2 A).

**Table 3:** Corneal astigmatism and surgically induced astigmatism estimated by vector analysis

	Non-Toric	Toric	*P* value†
Preoperative corneal astigmatism (D)			
Mean ± SD	-2.60 ± 0.94	-2.12 ± 0.43	0.27
95% CI	-1.88, -3.32	-1.79, -2.45	
Postoperative corneal astigmatism (D)			
Mean ± SD	-2.13 ± 1.09	-1.72 ± 0.71	0.48
95% CI	-2.97, -1.29	-2.26, -1.17	
Change in corneal astigmatism (D)			
Mean ± SE	0.47 ± 0.25	0.40 ± 0.25	
95% CI	-0.11, 1.05	-0.18, 0.99	
*P* values$	0.18	0.25	
Vector analysis, centroid@axis			
Preoperative corneal astigmatism	0.84 D@180°	0.84 D@173°	
Postoperative corneal astigmatism	0.73 D@5°	0.97 D@164°	
Surgically induced astigmatism	0.18 D @65°	0.29 D@137°	

†P values calculated by Mann-Whitney U test between non-toric and toric groups. $P values calculated by Wilcoxon’s signed-rank test between pre- and post-operative values in each non-toric or toric group. The postoperative values are collected 3 months postoperatively. SD, standard deviation; 95% CI, 95% confidence interval; SE, standard error; D, diopters.

## Discussion

In the current study, the postoperative UCVA was significantly better in the toric group than the non-toric group as a result of the lower refractive astigmatism in the former compared with the latter group. Larger postoperative refractive spherical error in the non-toric group than the toric group was explained by the compensation of the larger refractive astigmatism in the non-toric group, since the SEREs were not significantly different between the groups. Very recently, López-Caballero et al. reported the equivalent postoperative visual acuity and refractive status in toric IOL-implanted eyes between cataract surgery alone group and iStent inject triple procedure group [9]. Collectively, the results indicate the efficacy of toric IOLs to correct preoperatively existing corneal astigmatism in the glaucomatous eyes received simultaneous cataract surgery and iStent.

The SIA after iStent in this study seemed not different greatly from that after small-incisional cataract surgery (0.42 D after a 1.8-millimeter incision coaxial phacoemulsification and 0.5 D after 1.7-millimeter incision bimanual phacoemulsification).[16] Previously, reasons for SIA after LEC were reported to be a gap around the scleral flap [1], sinking of the limbus because of the tissue loss under the scleral flap [17], tissue contraction due to extensive scleral cautery [18], subconjunctival scarring [19, 20], corneal steepening due to the compression of a bleb by eyelid [18], and postoperative hypotony.[4] Absence of these possible mechanisms explains the small SIA by iStent [9] and other goniotomy-related MIGSs procedures [7, 8].

In the current study, the IOPs and the medications were unchanged postoperatively. Even in the larger scale randomized comparative study, the mean postoperative IOP was not significantly different between combined single iStent group and cataract surgery alone group [21]. IOP reduction after iStent was augmented by the increase in number of the stents implanted [22]. Rate of IOP reduction after trabecular bypass surgery was smaller in the eyes with low preoperative IOP [23]. The sample size of this study was calculated for the detection in refractive difference rather than detecting the IOP reduction. Accordingly, low statistical power due to small IOP reduction by single stent implantation in eyes with low preoperative IOP can explain the no statistically significant changes in IOPs and medications after the surgery in this study.

There are several limitations in this study including that the study was conducted by retrospectively, visual functions and status of refraction were analyzed at single postoperative time point, and the data about the misalignment of toric IOL axis was absent. It is possible that, if we included the long-term follow-up data, the results may alter, although we still believe that the study design of this study was scientifically reasonable to test the roles of toric IOLs on visual functions during early after the iStent triple procedures. Theoretically, implantation of iStent in combination of cataract surgery can associate with the increased chance of axis misalignment of toric IOL through the difficulty of alignment due to hyphema seen after iStent implantation and due to insufficient mydriasis frequently seen in glaucomatous eyes intraoperatively. Changes in anterior chamber depth and aqueous flow by implantation of iStent might have some impact on postoperative rotation of axis. These intra- and post-operative effects remains to be tested in the future. Given the eyes received MIGS can be treated by LEC later, thus the influence of previously-implanted toric IOL on the visual function after LEC is needed to explore in the future study.

In conclusion, use of toric IOLs is a reasonable option for better visual outcomes when the iStent was combined with cataract surgery in eyes with corneal astigmatism.

## Data Availability

The Full dataset for this study is available upon reasonable request to the corresponding author.
